# Glycosylated hemoglobin levels and the risk for contrast-induced nephropathy in diabetic patients undergoing coronary arteriography/percutaneous coronary intervention

**DOI:** 10.1186/s12882-021-02405-y

**Published:** 2021-06-02

**Authors:** H. Zhang, H. Fu, X. Fu, J. Zhang, P. Zhang, S. Yang, Z. Zeng, N. Fu, Z. Guo

**Affiliations:** 1 Clinical College of Chest,Tianjin Medical University, Tianjin, China; 2 Department of Cardiology, Tianjin Chest Hospital, No. 261, Taierzhuang South Road, Jinnan District, Tianjin, 300222 China; 3 Tianjin Medical University, Tianjin, China

**Keywords:** Diabetes mellitus, Glycosylated hemoglobin, Percutaneous coronary intervention, Contrast-induced nephropathy, Risk factors

## Abstract

**Backgrounds:**

Diabetes mellitus is an independent risk factor for Contrast-induced nephropathy (CIN) in patients undergoing Coronary arteriography (CAG)/percutaneous coronary intervention (PCI). Glycosylated hemoglobin (HbA1c) is the gold standard to measure blood glucose control, which has important clinical significance for evaluating blood glucose control in diabetic patients in the past 3 months. This study aimed to assess whether preoperative HbA1c levels in diabetic patients who received CAG/PCI impacted the occurrence of postoperative CIN.

**Methods:**

We reviewed the incidence of preoperative HbA1c and postoperative CIN in 670 patients with CAG/PCI from January 1, 2020 to October 30, 2020 and divided the preoperative HbA1c levels into 5 groups. Blood samples were collected at admission, 48 h and 72 h after operation to measure the Scr value of patients. Categorical variables were compared using a chi-square test, and continuous variables were compared using an analysis of variance. Fisher’s exact test was used to compare the percentages when the expected frequency was less than 5. Univariable and multivariable logistic regression analysis was used to exclude the influence of confounding factors, and P for trend was used to analyze the trend between HbA1c levels and the increased risk of CIN.

**Results:**

Patients with elevated HbA1c had higher BMI, FBG, and LDL-C, and they were more often on therapy with hypoglycemic agents, Insulin and PCI. They also had higher basal, 48 h and 72 h Scr. The incidence of CIN in the 5 groups of patients were: 9.8, 11.9, 15.2, 25.3, 48.1%. (*p* < 0.0001) The multivariate analysis confirmed that in the main high-risk subgroup, patients with elevated HbA1C levels (≥8.8%) had a higher risk of CIN disease. Trend test showed the change of OR (1.000,1.248,1.553,2.625,5.829).

**Conclusions:**

Studies have shown that in diabetic patients undergoing CAG/PCI, elevated HbA1c is independently associated with the risk of CIN, and when HbA1c > 9.5%, the incidence of CIN trends increase. Therefore, we should attach great importance to patients with elevated HbA1c at admission and take more active measures to prevent CIN.

## Background

Contrast-induced nephropathy (CIN) is reversible acute renal failure observed after administration of iodinated contrast media (CM) during angiographic or other medical procedures. It is defined as an increase of 25% or more, or an absolute increase of 0.5 mg/dl or more in serum creatinine (Scr) from baseline value, at 48 to 72 h following the exposure to CM [1, 2]. The exact mechanism of CIN is unclear, and it may be related to hemodynamic effects, the formation of reactive oxygen species (ROS), and renal tubular cytotoxicity [3]. In addition, studies have shown that inflammation, immune response, and the decrease in the number of endothelial progenitor cells (EPCs) will also affect the occurrence of CIN [4–6]. Many risk factors may contribute to the development of contrast nephropathy, such as age, glomerular filtration rate, preoperative hyperglycemia at blood cholesterol admission, and elevated glycosylated hemoglobin, both associated with CIN [7, 8]. The key to reducing the incidence of CIN is to identify patients at high risk of CIN and adopt appropriate prevention programs. Studies have found that patients with diabetes have a higher risk of CIN. Diabetic nephropathy has been identified as a decisive and independent risk factor for CIN [9]. Both diabetes and the administration of iodinated radiocontrast agents are associated with marked alterations of renal physiology, including changes in eGFR and renal hemodynamics, enhanced tubular transport activity and oxygen expenditure, and intensification of medullary hypoxia, and ROS generation [10]. Long-term hyperglycemia will cause many pathophysiological changes, such as endothelial and microvascular dysfunction, increased production of vascular inflammatory markers and ROS, and impaired immune response [11]. Measurements of glycosylated hemoglobin (HbA1c) can provide an average blood glucose level for the past 2–3 months and adequately reflect glycemic control in patients with diabetes [12]. Current studies have shown that among patients without diabetes undergoing CAG/PCI elevated HbA1c is independently associated with the risk of CIN [13]. However, there is no more data to confirm whether the level of HbA1c in patients with existing diabetic patients affects the occurrence of CIN. Therefore, compared with previous clinical trials, our study aims to focus on whether the level of HbA1c in diabetic patients undergoing CAG/PCI is related to the risk of CIN.

## Method

### Study population

**Fig. 1:**
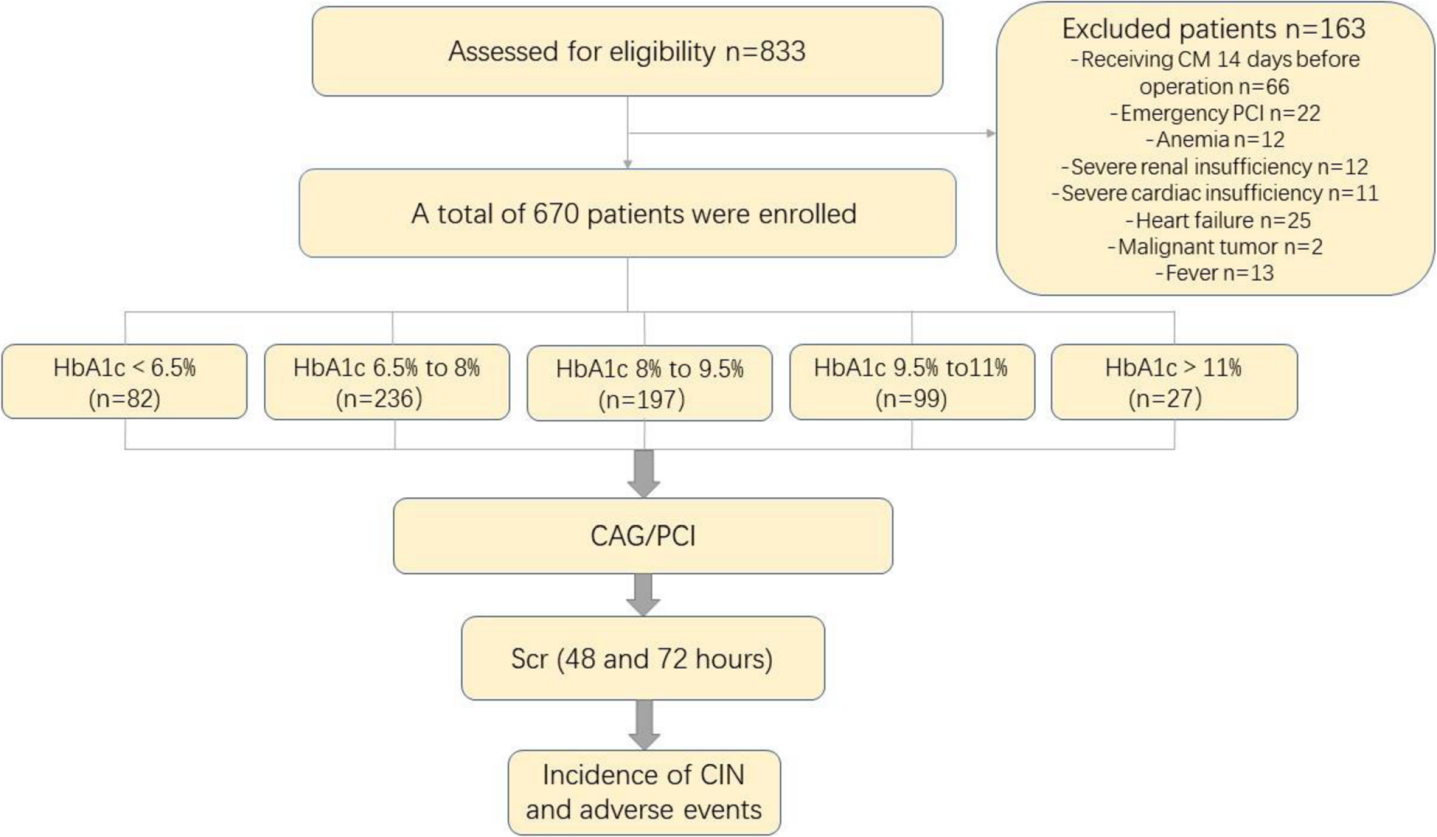
Patient flowchart. CAG: HbA1c: glycosylated hemoglobin; Coronary arteriography PCI; percutaneous coronary intervention; Scr: serum creatinine; CIN: contrast-induced nephropathy

We initially assessed 833 patients who underwent CAG/PCI. Inclusion criteria were as follows: Patients who meet the diagnostic criteria for diabetes and underwent CAG/PCI. [The American Diabetes Association (ADA) defines Criteria for the diagnosis of diabetes (1): fasting plasma glucose (FPG) > 126 mg/dL (7.0 mmol/L) (2); 2 h plasma glucose (PG) > 200 mg/dL (11.1 mmol/L) during oral glucose tolerance test (OGTT) (3); HbA1c > 6.5% (48 mmol/mol) (4); In a patient with classic symptoms of hyperglycemia or hyperglycemic crisis, a random plasma glucose> 200 mg/dL (11.1 mmol/L)] [14]. Exclusion criteria were acute ST-segment elevation myocardial infarction (STEMI) receiving emergency PCI, receiving CM 14 days before PCI, hypotension (systolic blood pressure < 90 mmHg), using any renal toxicity drugs during the perioperative period, severe renal insufficiency (creatinine clearance< 30 mL/min) or severe cardiac insufficiency (left ventricular ejection fraction (LVEF) < 30%), cardiogenic shock and heart failure, hypersensitivity to CM, severe liver damage, autoimmune diseases, malignant tumor, infectious diseases or fever. We also exclude other conditions that may affect HbA1c: hemoglobinopathy, pregnancy, uremia, blood transfusion, and hemolytic anemia [15]. (Fig. 1) The present study was approved by the Ethics Committee of the Tianjin Chest Hospital, and written informed consent was obtained from all participate before enrollment.

### Study protocol

We finally evaluated 670 diabetic patients undergoing CAG/PCI. The study protocol conforms to the ethical guidelines of the 1975 Declaration of Helsinki as reflected in a prior approval by the institution’s human research committee. Patients were stratified into 5 pre-procedural HbA1c groups: < 6.5%; 6.5 to 8%; 8 to 9.5%; 9.5 to 11%; > 11%. Because related studies have proved that hydration in the perioperative period is effective in preventing the occurrence of CIN [16, 17], all patients received isotonic saline (0.9% sodium chloride) 12 h before and after surgery and supplemented with standard hydration solution (at least 1000 mL) at a rate of 1 mL/kg/h. The hydration rate was reduced to 0.5 mL/kg/h for patients with LVEF⩽45%. All patients were given aspirin and clopidogrel loading dose 300 mg before surgery. Clinicians can decide whether to use the following medicines based on clinical requirements or guidelines, including b-blockers, angiotensin-converting enzyme inhibitors (ACEIs)/angiotensin II receptor blockers (ARBs), calcium channel blockers (CCBs), diuretics, and statin.

The risk of CIN is related to CM osmotic pressure. Iodine contrast agents include low-osmotic contrast media (LOCM), iso-osmotic contrast media, and high-osmotic contrast media [18]. Isotonic contrast agents are more effective than LOMC in reducing the incidence of CIN in patients [19]. Therefore, patients with normal and mildly abnormal Scr levels (Scr⩽177 μmol/L) before operation are treated with iopromide (LOCM). Compared with other CMs, iodixanol (isotonic contrast agent) has less nephrotoxicity, lower risk of cardiovascular adverse events, and thermal discomfort and is used in patients with moderately and severely abnormal Scr level (Scr > 177 μmol/L) [20, 21]. There was no statistically significant difference in the number of patients using contrast media between the five groups (*P* = 0.261).

### Study endpoints

Blood samples were collected at admission, 48 h, and 72 h after operation to measure the Scr value of patients. The primary study endpoint was CIN, diagnosed by the most elevated Scr concentrations 48 and 72 h after CM exposure. When the most elevated Scr is increased by 25% or more from the baseline value, or an absolute increase of 0.5 mg/dl or more, the diagnosis of CIN is established. Additional clinical endpoints included: Adverse events during hospitalization and 14-day follow-up, included all-cause mortality, hypotension or severe decrease in blood pressure, acute heart failure, coronary artery bypass, and graft cerebrovascular events.

### Statistical analysis

Results are expressed as numbers (%) or mean ± SD. Compare the number of cases or means between five groups: categorical variables were compared using a chi-square test, and continuous variables were compared using an analysis of variance. Fisher’s exact test was used to compare the percentages when the expected frequency was less than 5. Multivariable logistic regression analysis was used to exclude the influence of confounding factors, and to evaluate whether the association between pre-procedural HbA1c values and CIN persisted after adjustment for other patient characteristics and potential confounders. P for trend was used to analyze the trend between HbA1c levels and the increased risk of CIN. All statistical data was analyzed by SPSS software 22.0.

## Results

### Baseline clinical characteristics

There were no significant differences between the five groups in the baseline characteristics (Age, Male, Smoking, LVEF, Hypertension, Contrast volume, Hemoglobin, TG, TC, HDL-C, Hydration amount, Aspirin, Clopidogrel, b-blockers, ACEI/ARB, Diuretics, CCBs) before operation. Patients with elevated HbA1C levels had higher LDL levels (*p* = 0.010), BMI (*p* < 0.001), and Preoperative FBG (*p* < 0.001). In addition, patients with higher levels of HbA1C were more often on therapy with hypoglycemic agents (*p* = 0.033), Insulin (*p* = 0.011), and PCI. (*p* = 0.027) (Table 1).

**Table 1:** Comparisons of baseline characteristics between the five groups

Variables	HbA1c<6.5% (*n* = 82)	HbA1c6.5 to 8% (*n* = 236)	HbA1c8 to 9.5% (*n* = 197)	HbA1c9.5 to11% (*n* = 99)	HbA1c>11% (*n* = 27)	*P*
Age (years)	66.61 ± 7.13	67.78 ± 6.08	67.06 ± 7.42	66.55 ± 7.51	68.93 ± 5.78	0.296
Male (%)	43(52.4)	112 (47.5)	101 (51.3)	44 (44.4)	13 (48.1)	0.767
Smoking (%)	59 (72.0)	157 (66.5)	126 (64.0)	60 (60.6)	17 (63.0)	0.778
LVEF	61.12 ± 6.52	59.81 ± 7.29	60.50 ± 6.96	59.87 ± 7.31	61.63 ± 5.97	0.445
Hypertension (%)	40 (48.8)	129 (54.7)	118 (59.9)	65 (65.7)	16 (74.1)	0.140
BMI (kg/m^2^)	24.62 ± 2.28	24.90 ± 2.09	25.27 ± 1.85	25.63 ± 1.80	26.45 ± 1.70	< 0.0001
Contrast volume (mL)	168.90 ± 58.44	166.61 ± 52.07	170.91 ± 62.12	175.76 ± 70.34	175.19 ± 45.27	0.734
TG (mmol/L)	1.77 ± 1.02	1.75 ± 0.91	1.85 ± 1.10	1.75 ± 0.86	2.06 ± 0.87	0.512
TC (mmol/L)	4.55 ± 0.39	4.52 ± 0.41	4.61 ± 0.57	4.52 ± 0.52	4.57 ± 0.50	0.364
HDL-C (mmol/L)	1.30 ± 0.38	1.24 ± 0.33	1.28 ± 0.33	1.28 ± 0.40	1.26 ± 0.25	0.509
LDL-C (mmol/L)	2.46 ± 0.55	2.49 ± 0.61	2.50 ± 0.51	2.54 ± 0.49	2.89 ± 0.72	0.010
Preoperative FBG	6.63 ± 1.37	7.15 ± 1.51	7.95 ± 1.50	8.81 ± 1.54	10.09 ± 1.83	< 0.0001
Hydration amount (mL)	1305.5 ± 257.28	1322.4 ± 244.99	1277.4 ± 277.21	1278.8 ± 278.33	1305.6 ± 274.32	0.430
Aspirin (%)	77 (93.9)	220 (93.2)	191 (97.0)	92 (92.9)	25 (92.6)	0.451
Clopidogrel (%)	60 (73.2)	186 (78.8)	151 (76.6)	72 (72.7)	19 (70.4)	0.651
β-antagonist (%)	73 (89.0)	204 (86.4)	172 (87.3)	81 (81.8)	24 (88.9)	0.642
ACEI/ARB (%)	41 (50.0)	137 (58.1)	91 (46.2)	49 (49.5)	16 (59.3)	0.132
Diuretics (%)	7 (8.5)	20 (8.5)	13 (6.6)	7 (7.1)	2 (74.1)	0.950
CCB (%)	10 (12.2)	24 (10.2)	21 (10.7)	11 (11.1)	3 (11.1)	0.978
Hypoglycemic agents (%)	25 (30.9)	93 (39.4)	81 (41.1)	40 (40.4)	13 (48.1)	0.033^a^
Insulin (%)	6 (7.3)	19 (8.1)	20 (10.2)	16 (16.2)	7 (25.9)	0.011^a^
PCI (%)	45 (54.8)	141 (59.7)	124 (62.9)	69 (69.7)	23 (85.2)	0.027^a^
Basal Scr (mmol/L)	101.60 ± 15.03	102.14 ± 14.35	104.32 ± 11.48	109.94 ± 15.66	112.02 ± 12.45	< 0.0001
48 h Scr (mmol/L)	113.46 ± 16.46	114.18 ± 17.82	119.96 ± 15.67	127.42 ± 13.38	138.16 ± 9.74	< 0.0001
72 h Scr (mmol/L)	109.82 ± 16.25	109.01 ± 17.32	116.61 ± 14.47	119.29 ± 15.55	125.22 ± 13.84	< 0.0001
$\frac{48\mathrm{h}\ \mathrm{Scr}-\mathrm{basal}\ \mathrm{Scr}}{\mathrm{basal}\ \mathrm{Scr}}$	0.12 ± 0.09	0.12 ± 0.10	0.15 ± 0.13	0.18 ± 0.17	0.23 ± 0.14	< 0.0001
$\frac{72\mathrm{h}\ \mathrm{Scr}-\mathrm{basal}\ \mathrm{Scr}}{\mathrm{basal}\ \mathrm{Scr}}$	0.07 ± 0.07	0.09 ± 0.08	0.10 ± 0.07	0.12 ± 0.10	0.12 ± 0.11	< 0.0001

Data are expressed as mean ± SD or n (%). *ACEI* angiotensin-converting enzyme inhibitor, *ARB* angiotensin receptor blocker, *BMI* body mass index, *TC* total cholesterol, *TG* triglyceride, *HDL-C* high-density lipoprotein cholesterol, *LDL-C* low-density lipoprotein cholesterol, *LVEF* left ventricular ejection fraction, *CCB* calcium channel blockers, *FBG* fasting blood glucose, *PCI* Percutaneous Coronary Intervention

### Comparison of Scr level and CIN incidence in each group

Patients with elevated HbA1C had higher basal Scr, higher Scr values at 48 h and 72 h after CAG/PCI. We also compared the percent change in mean Scr compared with baseline creatinine at 48 and 72 h in 5 groups, and the difference was statistically significant. (*p* < 0.0001, Table 1) Univariate analysis was used to analyze the confounding factors that might be associated with CIN. (Table 2) Multivariate logistic regression analysis takes CIN as the dependent variable to exclude confounding factors and factors that may affect the development of CIN (male, age, LVEF, contrast volume, hydration amount, BMI, preoperative FBG, PCI, basal Scr, ACEI/ARB, diuretics, CCBs, HbA1c) were taken as independent variables. (HbA1c OR = 1.186(1.013–1.390), *p* = 0.034).

**Table 2:** Univariable logistic regression analyses analysis for certain confounding factors of CIN

Variables	Univariable analysis	
	OR (95% CI)	*P*
Age (years)	1.001 (0.971–1.032)	0.945
Male (%)	0.828 (0.543–1.262)	0.379
LVEF	1.032 (0.999–1.066)	0.057
Hypertension (%)	0.666 (0.423–1.050)	0.080
BMI (kg/m^2^)	1.146 (1.025–1.280)	0.016
Contrast volume (mL)	1.003 (0.999–1.006)	0.137
LDL-C (mmol/L)	0.652 (0.632–1.333)	0.652
Preoperative FBG	1.576 (1.361–1.825)	< 0.0001
Hydration amount (mL)	1.000 (0.999–1.001)	0.578
PCI (%)	4.031 (2.406–6.755)	< 0.0001
Basal Scr (mmol/L)	0.995 (0.980–1.010)	0.515

*LVEF* left ventricular ejection fraction, *BMI* body mass index, *LDL-C* low-density lipoprotein cholesterol, *FBG* fasting blood glucose, *PCI* Percutaneous Coronary Intervention

The trend test was used to further demonstrate the relationship between HbA1c levels and CIN incidence. It can be seen from the changes in Odd Ratio (OR) (1.000,1.248,1.553,2.625,5.829), with the increase of HbA1c level, the incidence of CIN has a significant increasing trend in the HbA1c 9.5–11 and > 11% groups compared with the HbA1c < 6.5% group, but there is no significant trend in the HbA1c 6.5–8 and 8–9.5 groups (Table 3).

**Table 3:** The logistic regression analysis and the trend test

HbA1c	Participants, n	CIN, n	Adjusted Odd Ratio		
			Model 1	Model 2	Model 3
<6.5%	82	9	1.000(Reference)	1.000(Reference)	1.000(Reference)
6.5 to 8%	236	28	1.450 (0.607–3.464)	1.453 (0.606–3.482)	1.248 (0.512–3.044)
8 to 9.5%	197	30	2.005 (0.844–4.759)	2.015 (0.846–4.800)	1.553 (0.643–3.804)
9.5 to11%	99	25	3.642 (1.483–8.945)	3.602 (1.459–8.894)	2.625 (1.025–6.718)
>11%	27	13	9.753 (3.268–29.114)	9.791 (3.260–29.409)	5.829 (1.785–19.034)
P for trend	–	–	<0.0001	<0.0001	<0.0001

Model 1 was adjusted for age and male. Model 2 was adjusted for age, male, hypertension, contrast volume and hydration amount. Model 3 additionally was adjusted for BMI, LDL-C and PCI

**Fig. 2:**
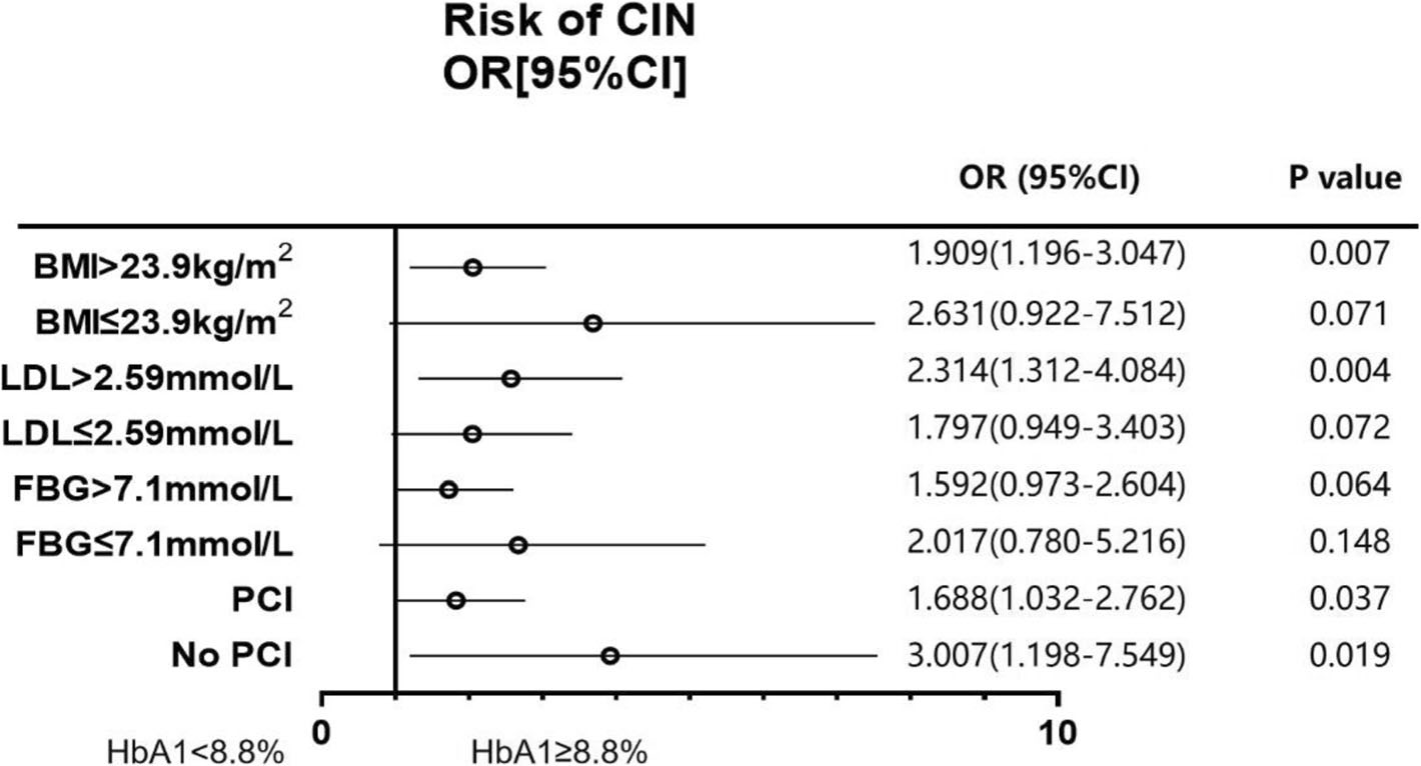
The association between HbA1c and CIN risk after adjusting for baseline confounding factors. 8.8% is the median elevated HbA1c levels. BMI: body mass index; LDL-C: low-density lipoprotein cholesterol; FBG: fasting blood glucose; PCI: Percutaneous Coronary Intervention

The multivariate analysis confirmed the association between HbA1c and the risk of CIN after adjustment for baseline confounding factors. The results showed that patients with elevated HbA1c (above the median value 8.75%) in the central high-risk subgroup had a higher risk of CIN, such as BMI (BMI > 23.9 kg/m^2^: adjusted OR = 1.909(1.196–3.047), *p* = 0.007), LDL (LDL > 2.59 mmol/L: adjusted OR = 2.314(1.312–4.084), *p* = 0.004), PCI (PCI: adjusted OR = 1.688(1.032–2.762), *p* = 0.037; No PCI: adjusted OR = 3.007(1.198–7.549), *p* = 0.019) (Fig. 2).

Major adverse events occurred in one patient (one hypotension) in group 1, four patients (one acute heart failure and one coronary artery bypass and two hypotension) in group 2, three patients (one acute heart failure two hypotension) in group 3, two patients (one graft cerebrovascular events and one coronary artery bypass) in group 4 and one patient (one hypotension) in group 5 during hospitalization and the 14 days of follow-up. There was no statistically significant difference in the incidence of major adverse reactions between the five groups during hospitalization and the 14 days of follow-up (*p* = 0.812).

## Discussion

In this study, we evaluated the relationship between preoperative HbA1c level and CIN incidence in patients undergoing CAG/PCI. This controlled trial showed that in diabetic patients undergoing CAG/PCI, elevated HbA1c is independently associated with the risk of CIN, and when HbA1c > 9.5%, the incidence of CIN trends increase. The results of multivariate analysis showed that the higher risk of CIN in patients with elevated HbA1C (> 8.8%) was confirmed in major high-risk subgroups, such as BMI > 23.9 kg/m^2^, LDL > 2.59 mmol/L, PCI, and no PCI. These results are of clinical significance, because with the improvement of PCI-related technology and equipment, the number of operations is increasing year by year, and the use of CM is becoming more and more common. CIN is a frequent complication after intravascular CM administration. It is the third most common cause of acute kidney injury in hospitalized patients, second only to ischemic and drug- induced injuries [22, 23]. The rat experiment showed that the CM-induced changes in diabetic rats indicate impaired renal function, oxidative stress, vascular dysfunction, and apoptosis. It was significantly higher in intensity compared to non-diabetic rats [24].

Before this, Stolker JM et al. have proven that for patients with known and unknown diabetes, elevated blood glucose levels before operation are a decisive and independent risk factor for CIN [25]. Barbieri L et al. have shown that HbA1c level is related to the risk of CIN occurrence in without diabetic patients [13]. Qin Y et al. found that the incidence of CIN in the elevated HbA1c group of patients undergoing CAG was higher than the group without elevated HbA1c [7]. We found that most previous studies mainly focused on admission blood glucose levels, representing the patient’s current blood glucose status and are affected by stress and recent diet [15]. Other studies included both diabetic and non-diabetic patients and were not targeted. Therefore, it is not clear whether HbA1c level will affect the occurrence of CIN in diabetic patients.

HbA1c measurement is a traditional method for evaluating blood sugar control. It reflects the average glucose concentration of red blood cells during the life cycle and can reflect the patient’s current blood sugar control status without being affected by external factors [26]. We focus on patients with type 2 diabetes (T2DM) because it is a significant risk factor affecting coronary artery disease (CAD), and 75% of T2DM patients die of cardiovascular disease [27–29]. Diabetes is an important predisposing factor for CIN, particularly in patients with renal function impairment. Renal hypoxia, combined with the generation of reactive oxygen species, plays a central role in the pathogenesis of CIN, and the diabetic kidney is particularly susceptible to intensified hypoxic and oxidative stress following the administration of contrast media. In brief, both diabetes and contrast agents enhance ROS formation. They also hamper renal oxygenation, either directly or through increased generation of ROS [10].

On this basis, we further studied whether HbA1c level also has an impact on CIN occurrence in diabetic patients and obtained favorable results. We hope that in the future, we can further explore whether preoperative intervention on HbA1c level can reduce the incidence of CIN, which will be of great significance for diabetic patients undergoing selective PCI, and a lot of well-designed experiments are needed.

### Limitations

Our research excluded patients with severe renal insufficiency, receiving CM 14 days before the operation, severe cardiac insufficiency, heart failure, malignant tumor, fever, and emergency PCI. We also excluded patients with anemia that may affect HbA1c. Although HbA1c remains the reference marker for assessing glycemic control and predicting the risk of development of long-term complications, it is limited. It cannot detect hypoglycemia or hyperglycemia daily and does not reflect rapid changes in day-to-day glucose control [12]. Further studies on the pathogenesis and preventive measures are needed to prevent CIN completely.

## Conclusions

Studies have shown that in diabetic patients undergoing CAG/PCI, elevated HbA1c is independently associated with the risk of CIN, and when HbA1c > 9.5%, the incidence of CIN trends increase. Therefore, we should attach great importance to patients with elevated HbA1c at admission and take more active measures to prevent CIN.

## Data Availability

The datasets used and/or analysed during the current study are available from the corresponding author on reasonable request.

## Abbreviations

HbA1c: Glycosylated Hemoglobin
CIN: Contrast-induced nephropathy
CAG: Coronary arteriography
PCI: Percutaneous coronary intervention
Scr: Serum creatinine
ACEI: Angiotensin-converting enzyme inhibitor;
ARB: Angiotensin receptor blocker
BMI: Body mass index
TC: Total cholesterol
TG: Triglyceride
HDL-C: High-density lipoprotein cholesterol
LDL-C: Low-density lipoprotein cholesterol
LVEF: Left ventricular ejection fraction
CCB: Calcium channel blockers
FBG: Fasting blood glucose
